# Polyprenyl Immunostimulant in Feline Rhinotracheitis: Randomized Placebo-Controlled Experimental and Field Safety Studies

**DOI:** 10.3389/fvets.2017.00024

**Published:** 2017-02-27

**Authors:** Alfred M. Legendre, Tanya Kuritz, Robert Eric Heidel, Vivian M. Baylor

**Affiliations:** ^1^Small Animal Clinical Sciences, College of Veterinary Medicine, University of Tennessee, Knoxville, TN, USA; ^2^Sass & Sass, Inc., Oak Ridge, TN, USA; ^3^Graduate School of Medicine, University of Tennessee, Knoxville, TN, USA; ^4^Independent Consultant, Oak Ridge, TN, USA

**Keywords:** feline rhinotracheitis, polyprenyl immunostimulant, feline herpesvirus, upper respiratory tract disease, clinical trials, treatment, clinical efficacy

## Abstract

Feline rhinotracheitis is a ubiquitous disease caused by feline herpesvirus type 1 (FHV-1). The disease is easily transmissible and common in multi-cat environments where even vaccinated cats can develop clinical signs of respiratory or ocular disease or both when exposed to the virus. Prior to the work reported here, there was no licensed treatment for the disease on the market. We hypothesized that polyprenyl immunostimulant (PI), an immunomodulatory veterinary biologic, would be useful in treating feline rhinotracheitis by reducing the severity of respiratory or/and ocular disease. We conducted double-blinded, randomized, placebo-controlled clinical trials in experimentally infected cats to establish the efficacy of PI. Specific pathogen-free cats were administered a placebo (*n* = 20) or PI (*n* = 20) starting on the day of FHV-1 experimental challenge. Trained, masked observers applied a standardized scoring system daily in clinical examinations for 14 days after the FHV-1 challenge. The cats treated with PI had significantly lower disease severity scores over the course of the experiment compared to the cats in the placebo group (*p* = 0.05). The safety studies, including a field safety study involving 390 owned cats in 10 states, showed that PI was safe to use in cats as young as 8 weeks of age.

## Introduction

Upper respiratory tract disease (URTD) is a common illness in cats that occurs worldwide. The principal agents of URTDs in domestic cats are feline herpesvirus type 1 (FHV-1) that causes rhinotracheitis and feline calicivirus (1). FHV-1 is highly contagious and easily spread between susceptible cats. URTD is a major cause of morbidity in multi-cat households such as breeding colonies, boarding catteries and shelters, and exposure of naïve cats to those environments leads to a high incidence of the disease (1, 2). FHV-1 causes respiratory or ocular disease or both that may range from mild to severe upper respiratory signs and systemic disease with lethargy, inappetence, and sneezing accompanied by nasal and ocular discharges. The acute infection may cause severe turbinate and nasal mucosal damage (3, 4) that might predispose the cats to lifelong bouts of bacterial nasal infection and sinusitis in sites of previous viral damage (1). During the course of an FHV-1 infection, the virus establishes itself in the trigeminal nerve ganglia (5). In times of stress and/or steroid administration, the latent herpesvirus may cause reactivation of respiratory and ocular signs and viral shedding (2). The persistence of the latent virus is usually lifelong (1, 6).

Treatment and control of FHV-1 infections in multi-cat households have been difficult. Adding lysine to the diet was beneficial in a research environment (7) but a recent study in a shelter environment found that supplementation with lysine may worsen the signs of FHV-1 infections (8). The treatment of acute infections with systemic interferon and antiviral drugs has been recommended in cats with FHV-1 infections. There was no benefit from ocular administration of feline recombinant interferon omega or human recombinant interferon alpha-2b on the clinical signs or viral shedding on cats with rhinotracheitis (9). Studies using famciclovir in a clinical trial and a series of case studies showed an antiviral benefit of the drug (10, 11), but it caused adverse events in 17% of the cats in the field application (12).

The herpesvirus disease can be controlled *via* cell-mediated immune response (1, 5). Polyprenyl immunostimulant (PI) is a veterinary biologic that modulates the immune response (13). We tested its ability to modulate and reduce the severity of the clinical disease. Our evaluation of clinical outcomes was based on the model that uses sign severity scores for nasal and ocular discharges on a scale from 0 (no disease) to 2 (severe disease) based on the veterinarian standard of practice.

## Materials and Methods

The studies presented here were carried out in compliance with U.S. Code of Federal Regulations (CFR) Title 9, and other laws governing research with veterinary biologics and animal studies at institutional, local, state, and federal levels. The studies were approved by the Institutional Animal Care and Use Committee of the University of Tennessee, protocols # 1946-0910 and 1654-0910. All cats were adopted after the completion of the efficacy studies.

### Safety Studies Design

The cats were monitored for the following adverse events defined by the Veterinary Dictionary for Drug Regulatory Activities, which included death, aggression, hyperactivity, vocalization, lymphadenopathy, abdominal pain, diarrhea, gastroenteritis, vomiting, anaphylaxis, angioedema, wheals, mouth rash or swelling, stinging or offensive taste, joint pain, muscle pain, lameness, ataxia, tremor, not drinking, anorexia, decreased appetite, general pain, weakness, depression, fever, lack of efficacy, loss of weight, poor feed conversion, tachypnea (other than panting), dyspnea, panting, sneezing, nasal discharge, cough, and ocular discharge. Each observer was provided the list of reportable events and directed to report their occurrence.

### Acute High-Dose Safety Study

Five specific pathogen-free (SPF) cats 12–14 weeks of age were randomly selected after the efficacy study. The cats were housed in the research facility in individual cages and were identified by ear tattoos. The cats had no signs of the disease. All cats were given 5 mg/kg (10× therapeutic) dose of PI orally and observed for 24 h for clinical signs of toxicity. No other interventions were provided.

### Field Safety Study

A field safety study was done in 2009 through 2010 in compliance with Title 9 of the CFR and approved by State Veterinarians of the 10 states where the study was conducted. The sample comprised 390 domestic cats of any breed, sex, and age. All cats without clinical disease established by their veterinarians and whose owners volunteered for the study were eligible. The study was overseen by veterinarians working with these cats.

Participating veterinarians were provided with PI, consent and observation forms. They were instructed on the product administration, record keeping, and reporting adverse events during and post-trials. The forms also included a preprinted list of reportable adverse events, and the owners and veterinarians were instructed to report those. PI was administered 0.5 mg/kg orally twice daily for at least 14 days by owners at their homes. The doses were calculated by the veterinarians. The times of medicating and observations were recorded daily by the owners and presented to veterinarians who validated the observations and returned the forms for evaluation.

### Efficacy Study Design

#### Participants and Eligibility Criteria

The studies were performed by the team at the University of Tennessee, College of Veterinary Medicine (UTCVM), the location closest to the manufacturer; the study Director Alfred M. Legendre and support staff have previously worked on PI evaluation. The study was done at the Veterinary Research Facility (VRF) at UTCVM in Knoxville, TN, USA. This is an air-conditioned indoor facility designed for small animal research and having appropriate facilities to prevent transmission of pathogens and provide isolation of study cohorts in closed rooms.

Specific pathogen-free cats were purchased for this study from Liberty Research Inc., Waverley, NY, USA, in 2007 (20 cats) and in 2013 (20 cats). All cats were tested for the lack of disease by the seller, and each one had a unique alphanumeric identifier (UID) tattooed inside the ears. Cats of any sex of 11–13 weeks of age with no clinical disease and having no prior exposure to the virus were considered eligible for inclusion. Case definitions are listed in Table 1.

**Table 1:** **Case definitions used for the outcomes on the study**.

Sign or test	Case definition	Clinical score or units
Ocular discharge	None	0
	Moderate (clear, serous)	1
	Severe (mucopurulent with crust formation)	2
Nasal discharge	None	0
	Moderate (clear, serous)	1
	Severe (mucopurulent with crust formation)	2
Nasal obstruction	None	0
	Moderate (some noisy nasal breathing)	1
	Severe (complete nasal obstruction)	2
Salivation	None	0
	Excessive salivation with visible saliva drooling from the mouth	1
Fever	Rectal temperature	°F
	>103.5	
	>104	
Weight change	Ratio of weight between days 0 and 14 day post-challenge (nominator) to weight at day 0 (denominator)	%
Anorexia (inappetence)	None (all wet food eaten up overnight)	–
	Any amount of wet food portion remained not eaten overnight	+
Dehydration	None	0
	Moderate by skin test (skin tenting evaluation—skin that is pulled up remains in place greater than 1 s and less than 2 s)	1
	Severe, requiring fluid therapy, skin remains in place longer than 2 s	2
Death or euthanasia	Reportable, necropsy required	Report
Feline herpesvirus type 1 (FHV-1) presence*	None	–
	Cytopathic effect observed in the Crandell-Rees feline kidney cell culture	+
FHV-1 antibody titer	None	0
	Titer reportable as minimal dilution upon direct titration of the sample	Dilution

### Interventions

All interventions were recorded on the daily Observation Forms for individual cats and on standardized intervention logs. During the interventions, the cats were housed in the same room in individual steel cases to which they were assigned at the intake.

Treatments were administered to individual cats by the university technicians. The volume of the treatment was calculated as 0.25 mL/kg of cat weight, and an appropriate volume was drawn from masked vials labeled with cats’ UIDs. Cats in the PI group received 0.5 mg/kg (0.25 mL/kg) of PI orally twice daily on days 0 through 14 post-challenge. The other group received an identical volume of placebo (0.25% aqueous *n-*butanol). The first dose was administered within hours after the challenge.

During the study, nasal and/or ocular discharges were cleaned at least once a day as needed. Subcutaneous fluids were administered as needed in case of severe dehydration.

Blood and oropharyngeal swabs were sampled for laboratory testing. The vials were coded with cats’ alphanumeric UIDs. A total of 3 mL of blood from the jugular vein of each cat was drawn before and after the challenge for measurement of FHV-1 antibody titers. Blood serum was separated and immediately shipped on ice to Washington State University for serology testing. Oropharyngeal swabs were taken before and after the challenge to identify the presence of virus. Immediately after sampling, oropharyngeal swabs were immersed into 2 mL of Dulbecco’s Modified Eagle Medium (DMEM) with 2% fetal bovine serum (FBS) and transported to the laboratory within the university campus. On day 0, all cats received a total of 1 mL of a 10^6^ TCID_50_/mL dose of FHV-1 by administering 1/3 mL in each nostril and 1/3 mL into the posterior oropharynx.

### Challenge

#### Viral Strain and Laboratory Procedures

All virology procedures were done at the Veterinary School Diagnostic Laboratory of the University of Tennessee (Knoxville, TN, USA), a certified laboratory. The FVR (FHV-1) strain SGE was obtained from the United States Department of Agriculture (USDA), Ames, IA, USA. The virus used in the challenge was grown and titrated by adding 15 μL aliquots of serial dilutions to Crandell-Rees feline kidney (CRFK) cell culture in 96-well culture plates with DMEM with 2% FBS. The virus titer was considered as the highest dilution of the culture that showed cytopathic effect (CPE). The challenge was adjusted to 10^6^ of 50% tissue culture infectious dose endpoint (TCID_50_) with minimal essential medium.

Each one of seven 25 cm^2^ flasks with CRFK cell culture was supplemented with 5 mL trypsin solution and incubated for 5–10 min at room temperature. The content was sterily transferred into 15 mL test tubes, and the cells were pelleted at 1,200 *g* for 2 min. The pellet was resuspended in 3 mL DMEM with 2% FBS, and equally split into three 25 cm^2^ flasks. Previously placed oropharyngeal swabs were removed from test tubes, and the content was mixed by vortexing, 1.5 mL was removed, syringe-filtered through 0.2 μm filter and added to the flasks with CRFK and incubated for 1 hour at room temperature. Then, 7 mL of DMEM with 2% FBS was added, and the flasks were transferred to the 37°C CO_2_ incubator. The flasks were inspected daily for 8 days, and results were recorded as CPE-positive (+) or negative (−).

Serology was done by a feline herpesvirus neutralization test at Washington Animal Disease Diagnostic Lab, SOP: 205.7.2010.08.07 (Washington State University, Pullman, WA, USA).

### Objectives

The objectives of this study were as follows: (1) to evaluate the safety of PI in clinically healthy cats of different signalments in a field study and (2) to evaluate the efficacy of the product in a randomized, blinded, placebo-controlled study with SPF cats experimentally challenged with FHV-1.

### Outcomes

Case definitions for all outcomes are listed in Table 1. The primary outcome was severity of the FRV disease measured based on standards of practice. Nasal discharge was considered as a measure of respiratory disease, and ocular discharge was used as a measure of ocular disease. The disease caused by FHV-1 may manifest as ocular or respiratory, or both, therefore, the scores for nasal and ocular discharges were combined in the case definition.

Secondary outcomes included fever defined as elevated rectal temperature over 103.5°F and over 104°F, average rectal temperature, weight change between the beginning and the end of the trial period, nasal obstruction, dehydration, salivation, and FHV-1 antibody titers.

All clinical assessments were performed by Alfred M. Legendre. All measurements and interventions followed the same protocol. The scales were calibrated daily using weight standards. Pictures of all cats were taken during all clinical evaluations. All laboratory tests were performed by certified veterinary diagnostic laboratories following standard procedures. After the trials, and before the code was broken, the remainder of the formulations in the used vials was analyzed for content by thin-layer chromatography, and the results were used to confirm the assignments by the pharmacy.

### Randomization Sequence Generation

Randomization occurred at three levels: (1) cats were assigned to cages in the random order they were unloaded from the truck and processed at the facility; the order of the assignments did not follow the order of UIDs listed on packing slip; (2) on 1 day before challenge (DBC) of each trial when the pharmacy at the UTCVM performed randomization and assigned treatments randomly based on the random-number table using UIDs listed on the packing slip; (3) during daily clinical observations, the order in which Alfred M. Legendre examined cats was randomized using www.randomizer.org.

### Blinding

The manufacturer provided labeled vials with PI and placebo (0.25% aqueous *n-*butanol) to the pharmacy at the UTCVM, which is located separately from the VRF. The pharmacy printed and affixed its own labels coded with the cats’ alphanumeric UIDs onto the vials corresponding to the treatment assignments. The pharmacy-labeled vials were given to technicians involved in the care of the cats. The firm, the researchers, or the technicians involved in the study did not participate in the treatment assignments. Pharmacy personnel did not participate in the care or evaluation of the cats and were blinded as to the cats’ assignments to cages.

During the study, the researchers and support personnel handling the cats and the laboratory samples used alphanumeric IDs corresponding to individual ear tattoos as identifiers. As an additional blinding measure to prevent pattern formation by Alfred M. Legendre, we applied randomization of the sequence of physical examinations of the cats using www.randomizer.org. Pharmacy personnel disclosed the key to the code to the investigators and support personnel after the end of the study.

### Statistical Analysis

Skewness and kurtosis statistics were used to test the assumption of normality for all modulation scores. A skewness or kurtosis statistic above an absolute value of 2.0 assumed a non-normal distribution. Levene’s Test of Equality of Variances was used to assess homogeneity of variance between the independent groups. If both statistical assumptions were met, then an independent samples *t*-test was used to compare control participants to treatment participants on continuous outcomes like modulation score, weight, and temperature. Means and SDs with 95% confidence intervals were reported for continuous outcomes. Chi-square tests were used to test associations between the treatment groups and categorical variables including gender, anorexia, mortality, and FHV-1 presence. Frequencies and percentages were reported for categorical outcomes.

The baseline clinical characteristics of the cats from the second trial were compared to those in the first trial using inferential statistics to confirm that the test subjects were virtually identical. These characteristics included weight, age, and gender. Independent samples *t*-tests were used for weight and age, while Chi-square was used for the gender comparison. Statistical significance was assumed at an alpha value of 0.05 and all analyses were conducted using SPSS Version 22 (IBM Corp., Armonk, NY, USA).

In our evaluation of the severity of the disease, which is the primary outcome, we used a composite score for two outcomes: nasal and ocular discharge. The severity of nasal and ocular discharges was used to denote modulation of disease states for each day of observation; the scores of 0 (no disease present), 1 (moderate disease), and 2 (severe disease) were added together to yield a composite score. Using this scoring methodology, the maximum total score for any given day of observation was 4 (severe for both ocular and nasal signs) and the minimum total score per day was 0 (no nasal or ocular discharge). With a total of 15 days of observation in this study, the highest score possible was 60 (the denominator). These modulation scores were added together for each day of observation (the numerator) and divided by 60 to yield the overall modulation score for each cat.

We compared extent of the severity of the disease in individual kittens between the PI and placebo groups. In that analysis, total scores per each kitten over the study period were calculated intragroup, and the distributions were compared between the PI and placebo groups using unpaired, two-tailed Student’s *t*-test.

Duration of the disease (persistence of the signs) was calculated as the number of days when at least one sign of the disease was present in a cat; the duration data were compared between the groups using unpaired, two-tailed Student’s *t*-test.

Chi-square tests were used to evaluate the significance of the intervention between the treatment groups with regard to dehydration where the total count of tests for dehydration over the observation period served as the denominator (300 for each group, the count of daily measurements in 20 cats over 15 days) and the count of recorded events (frequency) of dehydration served as a numerator for each treatment group. The same approach was used to evaluate fever ≥ 103.5°F and fever ≥ 104°F and salivation.

The unpaired, two-tailed Student’s *t*-test was used to evaluate the effect of treatment on FHV-1 antibody titers at the end of the trial [14 day post-challenge (DPC)], change in the body weight between days 0 and 14 DPC and average body temperatures.

## Results

### PI Is Safe

Polyprenyl immunostimulant was evaluated in an acute high-dose safety study on 26 September 2007 and a field safety study in 2009–2010 and found to be safe. In the acute high-dose safety study, the five SPF cats given a single 5 mg/kg oral dose (10× therapeutic dose) of PI had no adverse effects noted over the next 24 hours.

In the field safety study, observation forms reporting administration schedules and observations were collected from owners and veterinarians of 390 cats in 10 states and analyzed. The sample comprised 202 females and 188 males between 2 days and 16 years of age. The ages of cats on the study were: from 2 days to 4 weeks (53 cats, 13.6%), over 4 weeks to 8 weeks (75 cats, 19.2%), over 8 weeks to 6 months (121 cats, 31.0%), over 6 months to 3 years (96 cats, 24.6%), and over 3 years to 16 years (45 cats, 11.5%).

Three hundred fifty-nine (92%) of the cats received the PI for 14 or more days with the remaining 32 (8%) treated for less than 14 days. The reasons for protocol deviation included: death due to preexisting grave conditions (24, see Table 2), exacerbation of chronic colitis (1), owner’s discretionary decision to terminate their involvement in the study (2), cats were adopted (2), cat disliked the taste (1), cat developed diarrhea (1), caretaker had an insufficient supply of PI (1).

**Table 2:** **Reasons of death or euthanasia of the cats during or after the field safety study**.

State	Type of operation	Number of dead or euthanized cats	Reason
TX	No-kill rescue	18	Sixteen deaths of sick, abandoned kittens (2 days to 4 weeks of age), without queens, due to upper respiratory tract disease and malnourishment; one death due to an undiagnosed upper respiratory problem and one death in a cat infected with feline leukemia virus
AZ	Breeder	2	Effusive feline infectious peritonitis (FIP)
NE	Multiple-cat household	1	Non-effusive FIP
OH	No-kill rescue	3	Effusive FIP
Total		24	

Adverse events not attributed to PI included a total of 24 deaths because of preexisting grave conditions. Twenty-three cats died before the end of the treatment; one cat with advanced feline infectious peritonitis (FIP) died immediately after the end of the treatment. Table 2 summarizes the findings by the attending veterinarians. Five cats had effusive FIP when started on the PI, and one cat had advanced non-effusive FIP. Other adverse events during testing not attributed to PI toxicity were non-fatal. One owner noted that nine 7- to 9-week-old kittens from the same owner who had been previously weaned returned to nursing the queen after starting on the PI. One cat with chronic colitis before starting on the PI had a recurrence of colitis after starting PI. None of these events were considered by the USDA as an indication of toxicity from the PI.

Four adverse events were attributed to the PI. One cat had diarrhea, which started soon after the first dose of PI was given, and the PI was discontinued following the incident. Three cats exhibited a strong dislike for the taste or smell of PI. No other events were reported by the owners and veterinarians that were attributed to the PI.

### Efficacy Study

#### Study Flow

Specific pathogen-free cats identified with alphanumeric ear tattoos were delivered in individual crates to the UTCVM Research Facility. Upon unloading from the delivery truck, cats were randomly assigned to individual stainless steel cages in the same room and tagged with collars bearing tattoo ID and cage number. Each cat was given dry cat food and water *ad lib* and wet food once daily.

**Figure 1:**
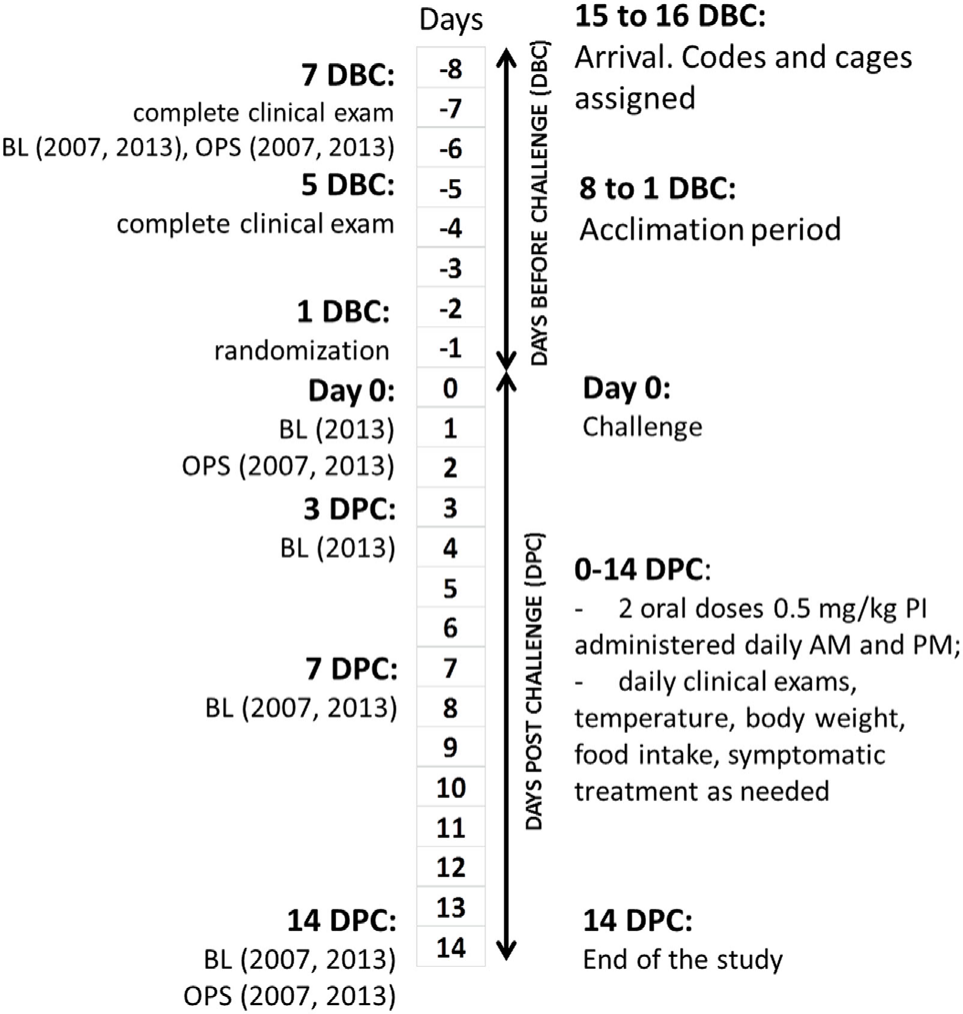
**Clinical study design and flowchart**. DBC, days before challenge; DPC, days post-challenge; BL, bleeding; OPS, oropharyngeal swabs.

The study flow is shown in Figure 1. All interventions, deviations, and observations were recorded daily on standardized forms, which were collected, reviewed, and signed by Alfred M. Legendre or technicians daily. Ear UIDs were validated every time each cat was handled for interventions or examinations. Laboratory test reports were filed together with the observation forms. Scales used in cat weighing were calibrated daily using weight standards. The cats were acclimated for at least 7 days before the experimental challenge (DBC; conditioning period). Cats were allowed to play together in the same room every day for 2–3 h during the conditioning period as well as during a 14-DPC period.

All cats were evaluated for the lack of clinical disease at 7 DBC (2007) or 8 DBC (2013), 5 DBC, and day 0. A total of 3 mL of blood from the jugular vein of each cat was drawn on 7 (2007) or 8 (2013) DBC, 0 (2013), 3 DPC (2013), 7 DPC, and on 14 DPC for measurement of FHV-1 antibody titers. Oropharyngeal swabs were taken on 7 DBC (2007) or 8 DBC (2013), 0 (2007), and 7 DPC.

The cats were challenged on day 0 and received a total of 1 mL of a 10^6^ TCID_50_/mL dose of FHV-1 by administering 1/3 mL in each nostril and 1/3 mL into the posterior oropharynx. Cats in the PI group received 0.5 mg/kg (0.25 mL/kg) of PI orally twice daily on days 0 through 14 post-challenge. The other group received an identical volume of placebo (0.25% aqueous *n-*butanol). The first dose was administered within hours after the challenge. The cats were clinically evaluated daily after the experimental FHV-1 challenge until 14 DPC. The same clinician (Alfred M. Legendre) did the physical examinations in a random sequence. The exams included measurement of weight and rectal temperature, scoring the severity of nasal discharge and nasal obstruction, and scoring the severity of ocular disease by ocular discharge. Dehydration and salivation were also scored.

During the study, nasal and/or ocular discharges were cleaned at least once a day. If case of severe dehydration (Score 2), subcutaneous fluids were administered.

All cats were seronegative prior to the experimental challenge and seropositive after the challenge.

### Dates Defining Periods of the Study

The study was conducted from 4 September 2007 (day 0, inoculation) through 25 September 2007 (last day of daily observations) and from 15 January 2013 (Day 0) through 30 January 2013 (last day of daily observations). After the end of daily observations, the cats remained at the research facility and were observed periodically until all cats cleared signs, were vaccinated, neutered, and adopted. Twenty cats were used in each trial for a total of 40 cats. Every attempt was made in the 2013 study to replicate exactly the 2007 study. The same study protocol was used by the same research team at the University of Tennessee.

### Baseline

Both parts of the study involved cats of the same age from the same source: domestic shorthair SPF. Baseline characteristics of treatment cohorts are presented in Table 3. All cats (20/20 in 2007 and 20/20 in 2013) were 11–13 weeks of age and clinically healthy at the three prechallenge evaluations. Prechallenge tests for virus isolation from oropharyngeal swabs and serology indicated the lack of the virus and FHV-1 and antibodies in all 40 cats. All 40/40 cats met the inclusion criteria.

**Table 3:** **Baseline characteristics of cat cohorts in the polyprenyl immunostimulant efficacy trials**.

	2007		2013	
Variable	Treatment	Placebo	Treatment	Placebo
*n*	10	10	10	10
Weight at day 0, g (M ± SD)	1,400.7 ± 145.9	1,424.6 ± 196.7	1,359.9 ± 119.8	1,277.9 ± 104.0
Age at day 0, days (M ± SD)	90.4 ± 2.5	90.4 ± 2.5	78.3 ± 3.5	79.9 ± 5.1
Sex, *n*				
Male	5	5	5	7
Female	5	5	5	3

### Numbers Analyzed

All cats completed the assigned treatments; 20/40 cats received placebo and the other 20/40 were treated with PI as intended. All cats enrolled into the study survived, and all data were collected.

### Outcomes

After the inoculation, all 40 cats developed nasal discharge. Ocular discharge was observed in 13/20 cats in the PI group and 15/20 cats in the placebo group. One cat in the placebo group had a mild nasal discharge throughout the whole study, whereas one cat in the PI group was severely affected. The daily clinical scores for nasal and ocular discharges were added and compared between groups. Table 4 shows summary statistics for the outcomes in both groups.

**Table 4:** **Outcome statistics summary**.

Outcome	Units	Treatment group	Placebo group	*p-*Value
Severity of rhinotracheitis	Modulation scores	0.21 ± 0.12	0.30 ± 0.15	0.05*
Duration of rhinotracheitis (persistence of the disease)	Days	7.65 ± 2.39	9.35 ± 3.03	0.06
Individual disease severity	Severity scores	12.65 ± 6.88	17.70 ± 8.77	0.05*
Weight change
Day 0	g	1,380.30 ± 131.59	1,351.25 ± 170.63	0.41
14 day post-challenge (DPC)		1,421.8 ± 205.41	1,431.45 ± 259.14
Average body temperature	°F	101.95 ± 1.02	101.99 ± 1.13	0.55
Fever ≥39.7°C (103.5°F)	Frequency	25	32	0.9
Fever ≥40°C (104.0°F)	Frequency	11	21	0.07
Feline herpesvirus type 1 antibody titer on 14 DPC	Titer	0.12 ± 0.07	0.12 ± 0.08	0.73

*Outcome definitions, unit descriptions, and CI 95% intervals are in the text*.

*Statistical significance.

There was no significant difference in antibody titers, expressed as dilutions, between the treatment group (M = 0.12, SD = 0.07) and the placebo group (M = 0.12, SD = 0.08) measured on 14 DPC, *p* = 0.73. No virus was identified on day 8 pre-challenge from oropharyngeal swabs from cats, whereas FHV-1 was isolated from all oropharyngeal swabs taken on 7 DPC confirming that all cats in the study were infected with the FHV-1.

**Figure 2:**
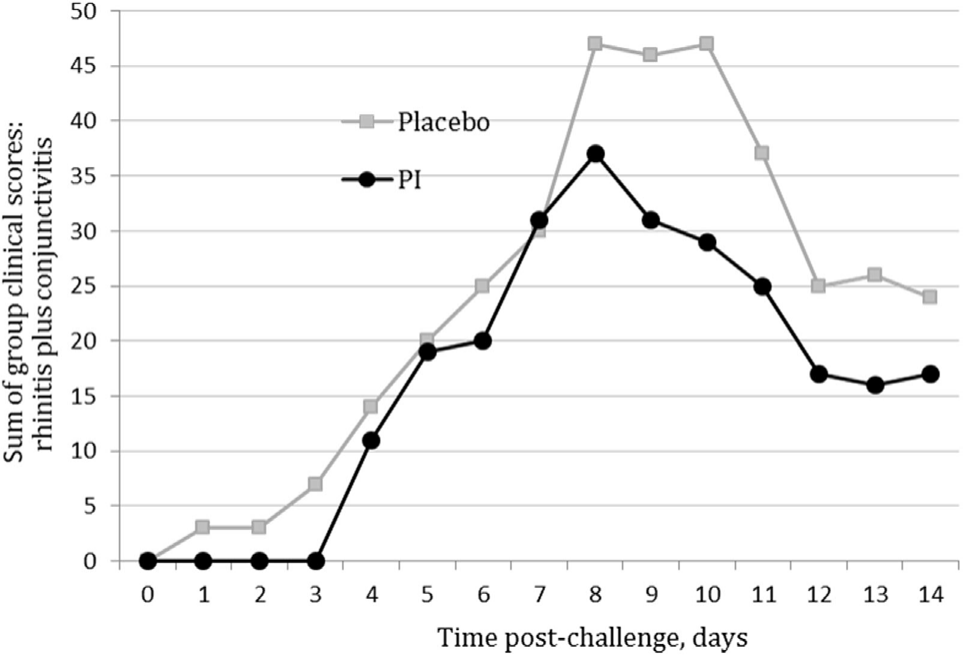
**Severity scores for nasal and ocular discharge in the polyprenyl immunostimulant (PI)-treated (*n* = 20) and placebo (*n* = 20) groups**. Control participants had significantly higher modulation scores over the course of the experiment in comparison to treatment participants, *p* = 0.05 (independent samples *t*-test).

**Figure 3:**
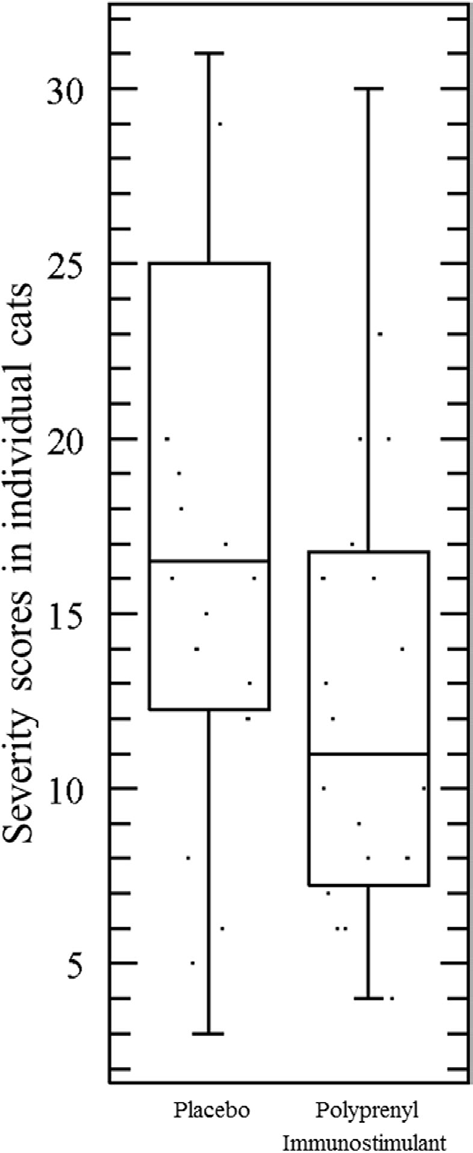
**Total severity scores in individual cats were significantly lower in the cats treated with polyprenyl immunostimulant (*n* = 20) compared to the cats treated with placebo (*n* = 20), *p* = 0.05, unpaired, two-tailed Student’s *t*-test**. Each point represents total severity scores of an individual cat during 0–14 DPC.

**Figure 4:**
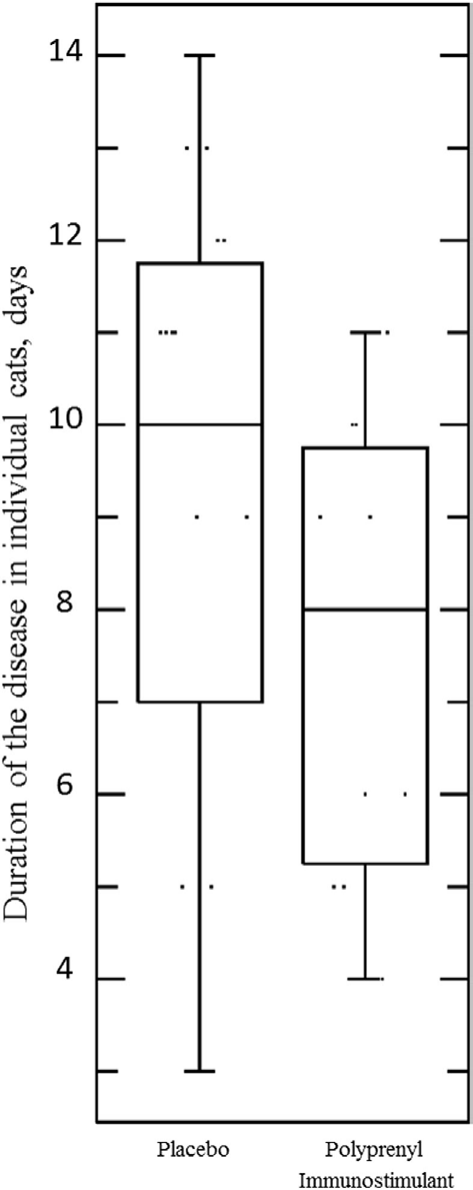
**Duration of the disease (persistence of ocular and/or nasal discharge) in individual cats in the Pi-treated (*n* = 20) and placebo (*n* = 20) groups was not significant, *p* = 0.06, unpaired, two-tailed Student’s *t*-test**. Each point represents the number of days when an individual cat presented with either or both signs during 0–14 DPC.

The assumptions of normality and homogeneity of variance were met for the between-subjects comparison of control participants to treatment participants; Figure 2 shows the dynamics of the clinical scores for the disease severity over the duration of the study. Treatment participants had significantly higher modulation scores (M = 0.21, SD = 0.12) over the course of the experiment in comparison to control participants (M = 0.30, SD = 0.15), *p* = 0.05, 95% confidence interval [95% CI] of difference 0.00003–0.1683, η^2^ = 0.10, power = 0.52. Total severity scores for individual cats were significantly lower in the PI-treated group compared to placebo (*p* = 0.05, 95% CI of difference 0.0021–10.10, η^2^ = 0.10, power = 0.51; Figure 3). No significant difference was observed between the groups in the duration of the persistence of the signs (*p* = 0.06; Figure 4).

A total of 73% (29/40) of the cats had fever at least once during the 14 days after the virus challenge. Fever equal to or greater than 39.7°C (103.5°F) was recorded 25/300 times in the PI in experimental and 32/300 in placebo groups [odds ratio (OR) = 0.76, 95% CI = 0.44–1.32]. The difference was not statistically significant, *p* = 0.34 (chi-square test). Fever of ≥40.0°C (104.0°F) was observed in 11/300 in the PI group and in 21/300 in the placebo group (OR = 0.53, 95% CI 0.25–1.11). The difference between frequencies of observations was not statistically significant, *p* = 0.07 (chi-square test). The difference between average body temperatures also lacked significance, *p* = 0.55, η^2^ = 0.02, power = 0.29 (unpaired, two-sided Student’s *t*-test).

Over the 14 days after challenge, the mean and SD of the cat’s weight went from 1380.30 ± 131.59 g to 1421.80 ± 205.41 g in the PI group and 1351.30 ± 170.63 g to 1431.45 ± 259.14 g in the placebo group. The differences were not statistically significant, *p* = 0.41, η^2^ = 0.03, power = 0.28 (unpaired, two-sided Student’s *t*-test).

Dehydration of various degrees was observed in seven cats in the placebo and eight cats in the treatment group; however, the frequencies did not differ significantly. Seven cats in the PI group required fluid therapy for a total of nine applications and five cats in the placebo group required a total of eight applications of fluid therapy for dehydration. The difference was not statistically significant. Severity scores of salivation did not differ significantly between the groups.

Our intent to analyze modulation of nasal obstruction was unsuccessful because the sign was transient and changed after sneezing or activity. Therefore, we are not reporting any statistical findings on this outcome.

No cat in the study developed mouth ulcerations. No animals died or were euthanized during the trial. After the trial, all animals were either adopted to individuals (35/40) or remained at UTCVM colony (5/40).

### Adverse Events

No adverse events from the use of PI were noted throughout the study.

## Discussion

Feline herpesvirus-1 infection is a common cause of respiratory disease in cats worldwide. Control of the herpesvirus disease requires support of the immune response where severity of signs and time to resolution depend on the equilibrium between viral evasion of the immune system and effective immune protection (14). We hypothesized that control of signs of feline rhinotracheitis can be achieved through modulation of the immune response and therefore PI, an immunomodulator, should decrease the severity of the clinical disease.

Feline herpesvirus type 1 can produce severe illness especially in young cats and presents a higher threat to younger cats and kittens; therefore, in our safety study, we specifically focused on the safety in younger kittens, and 128/390 cats (32.8%) on our study were under 8 weeks of age. Adverse reactions in 4/390 cats (1%) included an immediate onset of diarrhea (1) and an active dislike of the taste or smell of the PI (3). Whereas the intent was to assess safety in normal cats/kittens, a number of the participants tried PI in their sick cats. The 24 lethal events reported as adverse on the evaluation forms were due to preexisting diseases and not considered PI-related (Table 2), including FIP (6/24), feline leukemia virus infection (1/24), an undiagnosed, preexisting severe respiratory problem (1/24), and malnourishment in neonatal, orphaned kittens (16/24). PI caused no adverse events at the 10× therapeutic dose in the acute toxicity study.

The experimental study was designed as randomized, double-blinded, placebo-controlled and involved SPF cats challenged with a standard strain used in the development and evaluation of rhinotracheitis vaccines by other groups (5, 15). The challenge followed by an immediate treatment was similar to the clinical trials with famciclovir (10).

The trial was intended as an efficacy study for licensing of PI by the USDA and was conducted under the legal requirements for clinical trials. Those requirements [reviewed by Kleist (16)] directed the selection of the outcomes and the scoring system. In line with the regulations, our definition of clinical disease included two disease-defining: upper respiratory disease scored by the severity of nasal discharge and ocular disease scored by the severity of ocular discharge. The scoring of each sign was limited to 0–1–2 (no sign–moderate–severe) to allow unequivocal and reproducible evaluation based on the obvious differences between serous and mucopurulent discharges.

Individual variability in the severity of the signs within the groups was the reason we did not see a significant difference between the scores in the groups in our first trial in 2007 (*p* = 0.09); there was insufficient statistical power of the sample (too few cats). Twenty cats were considered the maximum number of cats that could be reasonably studied at one time with the available resources, e.g., room size, team size, and evaluation requirements. US regulations allow combining data from separate clinical trials provided that the trials are similar. Our two challenge experiments were virtually identical with respect to protocol, therapeutic dose, number of cats in the groups, age of cats, challenge regimen, and research team. The cats came from the same source, and there was no statistically significant difference between signalments in the cat samples in both studies. Furthermore, the course of the disease in the groups in both studies was identical. The difference in the scores between the groups was significant (*p* = 0.05) after the data from the two trials were combined. USDA accepted the data from the combined trials and licensed PI.

The signs of upper respiratory and ocular disease have been extensively used as markers of the efficacy of vaccines designed to protect against rhinotracheitis (1–4, 15, 17, 18). We used the composite score for the disease comprising two primary, measurable outcomes. The composite score did not include secondary outcomes such as nasal obstruction or non-specific signs such as fever, anorexia, dehydration, and salivation. The effect of PI on secondary outcomes (i.e., non-specific signs) was analyzed separately, for each sign, as required by the regulations (16), and no significant difference in the severity of secondary outcomes was found between the treatment and placebo groups.

In our studies, we used a scale similar to vaccine and drug efficacy studies. Serous nasal and ocular discharges were considered as less severe (score of 1) than mucopurulent discharges [score of 2; Ref. (12, 15, 18, 19)]. The absence of a sign received a score of 0. While nasal obstruction and sneezing are commonly mentioned as signs of the disease, we excluded those from our analysis because those signs are highly variable over short periods of time, making it difficult to grade them consistently.

All cats in both groups developed signs of FHV-1 infection at some time in the study, and the progress of the disease was as described by others (15, 18, 19). We restricted our analysis to days 0–14 DPC, which is the well-recognized duration of the virus-induced phase of the disease (1). Even in SPF cats of the same age and infected with the same strain of the FHV-1 virus, severity of the clinical signs varied a great deal within both groups in our study, in line with those previously reported in natural (1) and experimental (17) infections. One cat in the placebo group developed only minimal signs, two kittens in the placebo, and two kittens in the PI group developed an extremely severe disease.

The overall analysis identified a statistically significant decrease in severity of the nasal and ocular signs of rhinotracheitis in the group treated with PI compared to placebo-treated cats in spite of considerable individual variations in both groups (*p* = 0.05). PI also reduced summary scores for individual kittens in the treated group compared to placebo (*p* = 0.05). No significant difference was observed between the groups in the duration of the persistence of the signs (*p* = 0.06), although the result may be due to the insufficient sample size.

Reducing the severity of clinical signs is beneficial in improving the quality of life of the cats while they are recovering from FHV-1 infection and may be associated with a reduction of the epithelial necrosis and the damage to the turbinates that occurs with herpesvirus, thereby reducing the long-term sequelae such as chronic nasal infections and sinusitis. FHV-1 replicates in the nasal turbinates (1, 3) and can cause permanent damage. Damaged turbinates provide an area for secondary bacterial infections that can cause permanent signs of rhinitis.

Treatment of rhinotracheitis has been difficult and has consisted mainly of supportive care such as maintaining hydration and electrolyte balance, cleaning nasal discharges, broad spectrum antibiotics to prevent secondary infection, and encouraging eating (3). Recent studies done in a shelter environment noted worsening of the respiratory signs in cats supplemented with lysine (8). Earlier studies with valacyclovir found it to be too toxic for use in cats with FHV-1 infections (20).

Recently, a number of groups reported the use of famciclovir, an antiviral drug (10). In the comparable model with the experimental infection, Thomassy et al. (11) demonstrated that in the experimental infection, famciclovir reduced viral load and severity of the disease. We cannot make a direct comparison between the efficacy of PI and famciclovir because the scoring was done in very different ways. The clinical efficacy of famciclovir was evaluated by a composite score of five primary and secondary outcomes: the sum of interrelated endpoints consisting of upper respiratory signs (sneezing and nasal discharge) and interrelated ocular signs (conjunctivitis, blepharospasm, and ocular discharge), with more scoring grades, and a potential highest score of 24 versus the highest potential score of 4 in our study. While the use of composite scores and the increase in the number of scoring grades adds power to the sample and requires fewer cats and fewer observation points, we chose not to combine primary and secondary and interrelated outcomes because this method may affect the validity of the conclusions (21). Based on our safety studies, PI was much safer than famciclovir, which caused adverse reactions in 17% of the treated cats (12).

Polyprenyl immunostimulant reduces clinical severity of the disease probably through immunity upregulation but has no effect on viral or antibody titers. Because of the dosage frequency and its application as an oral liquid, PI is easier to administer than pills. Simultaneous treatment with antiviral and immunomodulatory compounds has been successfully used in human medicine for HIV, hepatitis C, and other diseases and may be an option in the management of feline rhinotracheitis. PI and famciclovir represent two separate classes of treatments and both appear to reduce the severity of FHV-1 infections. No studies have been done to see if the famciclovir and PI effects can complement each other.

## Author Contributions

AL: conception and design of the work, interpretation of the data, revising the manuscript critically for important intellectual content, and final approval of the version to be published. TK: post-study acquisition of the data, analysis and interpretation of data for the work, drafting the work and revising it critically for important intellectual content, and final approval of the version to be published. RH: data analysis and interpretation, revising the manuscript critically for important intellectual content, and final approval of the version to be published. VB: data acquisition, entry and organization, revising the data and the manuscript critically for important intellectual content, and final approval of the version to be published. All the authors agreed to be accountable for all aspects of the work in ensuring that questions related to the accuracy or integrity of any part of the work are appropriately investigated and resolved.

## Conflict of Interest Statement

AL, VB, and RH do not have a financial interest in Sass & Sass, Inc. TK is an employee and a minor stakeholder in Sass & Sass, Inc. VB and RH were consultants to Sass & Sass. No financial incentives were provided to owners and veterinarians participating in the study.

## References

[1] Gaskell RN Dawson S Radford A Feline respiratory disease. 4th ed In: Greene CE, editor. Infectious Diseases of the Dog and Cat. St. Louis: Elsevier Saunders (2012). p. 151–64.

[2] Hickman MA Reubel GH Hoffman DE Morris JG Rogers QR Pedersen NC. An epizootic of feline herpesvirus type 1 in a large specific pathogen-free cat colony and attempts to eradicate the infection by identification and culling of carriers. Lab Anim (1994) 28:320–9. 10.1258/002367794780745038 7830371

[3] Thiry E Addie D Belák S Boucraut-Baralon C Egberink H Frymus T Feline herpesvirus infection ABCD guidelines on prevention and management. J Feline Med Surg (2009) 11:547–55. 10.1016/j.jfms.2009.05.003 19481034 PMC7129359

[4] Cocker FM Newby TJ Gaskell RM Evans PA Gaskell CJ Stokes CR Response of cats to nasal vaccination with a live, modified feline herpesvirus type 1. Res Vet Sci (1986) 41:323–30. 3027798

[5] Sussman MD Maes RK Kruger JM. Vaccination of cats for feline rhinotracheitis results in a quantitative reduction of virulent feline herpesvirus-1 latency load after challenge. Virology (1997) 228:379–82. 10.1006/viro.1996.8393 9123845

[6] Maes R Feline herpesvirus type 1 infection in cats: a natural host model for alphaherpesvirus pathogenesis. ISRN Vet Sci (2012) 2012:14 10.5402/2012/495830 PMC3671728 23762586

[7] Stiles J Townsend WM Rogers QR Krohne SG. Effect of oral administration of l-lysine on conjunctivitis caused by feline herpesvirus in cats. Am J Vet Res (2002) 63:99–103. 10.2460/AJVR.2002.63.99 16206789

[8] Drazenovich TL Fascetti AJ Westermeyer HD Sykes JE Bannasch MJ Kass PH Effects of dietary lysine supplementation on upper respiratory and ocular disease and detection of infectious organisms in cats within an animal shelter. Am J Vet Res (2009) 70:1391–400. 10.2460/ajvr.70.11.1391 19878022

[9] Slack JM Stiles J Leutenegger CM Moore GE Pogranichniy RM. Effects of topical ocular administration of high doses of human recombinant interferon alpha-2b and feline recombinant interferon omega on naturally occurring viral keratoconjunctivitis in cats. Am J Vet Res (2013) 74:281–9. 10.2460/ajvr.74.2.281 23363355

[10] Malik R Lessels NS Webb S Meek M Graham PG Vitale C Treatment of feline herpesvirus-1 associated disease in cats with famciclovir and related drugs. J Feline Med Surg (2009) 11:40–8. 10.1016/j.jfms.2008.11.012 19154974 PMC11135480

[11] Thomassy SM Lim CC Reilly CM Kass PH Lappin MR Maggs DJ. Evaluation of orally administered famciclovir in cats experimentally infected with feline herpesvirus type-1. Am J Vet Res (2011) 72:85–95. 10.2460/ajvr.72.1.85 21194340

[12] Thomassy SM Shull O Outerbridge CA Lim CC Freeman KS Strom AR Oral administration of famciclovir for treatment of spontaneous, ocular, respiratory, or dermatologic disease attributed to feline herpesvirus type I: 59 cases (2006-2013). J Am Vet Med Assoc (2016) 249:526–35. 10.2460/javma.249.5.526 27556267

[13] Danilov LL Deeva AA Kuritz T Maltsev SD Narovlianskiy AN Pronin AV Therapeutic Composition and Methods. Washington, DC: U.S. Patent and Trademark Office (2003). U.S. Patent No 6,525,035.

[14] Melchjorsen J Matikainen S Paludan SR. Activation and evasion of innate antiviral immunity by Herpes simplex virus. Viruses (2009) 1:737–59. 10.3390/v1030737 21994567 PMC3185509

[15] Scott FW Geissinger CM. Long-term immunity in cats vaccinated with an inactivated trivalent vaccine. Am J Vet Res (1999) 60:652–8. 10328440

[16] Kleist P Composite Endpoints: Proceed with Caution. Applied Clinical Trials (2006). Available from: http://www.appliedclinicaltrialsonline.com/composite-endpoints-proceed-caution?id=&sk=&date=&%0A%09%09%09&pageID=2

[17] Gaskell RM Povey RC The dose response of cats to experimental infection with feline viral rhinotracheitis virus. J Comp Pathol (1979) 89:179–91. 10.1016/0021-9975(79)90057-4 222816

[18] Povey RC Koonse H Hays MB Immunogenicity and safety of an inactivated vaccine for the prevention of rhinotracheitis, calicivirus disease and panleukopenia in cats. J Am Vet Med Assoc (1980) 177:347–50. 6256329

[19] Orr CM Gaskell CJ Gaskell RM Interaction of an intranasal combined feline viral rhinotracheitis, feline calici vaccine and the FVR carrier state. Vet Rec (1980) 106:164–6. 10.1136/vr.106.8.164 6244692

[20] Nasisse MP Dorman DC Jamison KC Weigler BJ Hawkins EC Stevens JB Effect of valacyclovir in cats infected with feline herpesvirus-1. Am J Vet Res (1997) 58:1141–4. 9328668

[21] Cordoba G Schwartz L Woloshin S Bae H Gøtzsche PC. Definition, reporting, and interpretation of composite outcomes in clinical trials: systematic review. BMJ (2010) 341:c3920. 10.1136/bmj.c3920 20719825 PMC2923692

